# Reductions in Hospital Admissions and Delays in Acute Stroke Care During the Pandemic of COVID-19

**DOI:** 10.3389/fneur.2020.584734

**Published:** 2020-11-05

**Authors:** Yiqun Wu, Fei Chen, Zijing Wang, Wuwei Feng, Ying Liu, Yuping Wang, Haiqing Song

**Affiliations:** ^1^Department of Epidemiology and Biostatistics, School of Public Health, Peking University Health Science Center, Beijing, China; ^2^Department of Neurology, Xuanwu Hospital, Capital Medical University, Beijing, China; ^3^Department of Neurology, Duke University School of Medicine, Durham, NC, United States; ^4^Beijing Municipal Health Commission, Beijing, China; ^5^Beijing Stroke Quality Control Center, Beijing, China

**Keywords:** stroke, acute care, COVID-19, hospital admission, quality of care

## Abstract

**Background:** Rapid and effective medical care for stroke is paramount to achieve maximal functional recovery. Because of the wide spreading of the coronavirus disease in 2019 (COVID-19), acute stroke care is negatively impacted. How much acute care for stroke has been affected during the pandemic remains to be assessed.

**Methods:** The first-level response to major public health was launched from January 24th to April 29th, 2020 in Beijing to contain the spread of COVID-19. Based on a database connecting all 77 stroke centers, the quantity and quality in emergency care for stroke during the 97 lockdown days were compared with the equivalent period in 2019. During the pandemic, 15 of the 77 stroke centers were designated to receive patients sick with COVID-19. Subgroup analyses were carried out by different types of hospitals (designated and undesignated).

**Results:** There were 1,281 and 2,354 stroke emergency hospital admissions in the lockdown period and the parallel period in 2019, respectively. A reduction of 45.6% in admission was shown in the lockdown period, with more reductions for hemorrhagic stroke (69.0%) compared with ischemic stroke (42.9%). More reductions happened in COVID-19 designated hospitals (52.6%) compared with undesignated hospitals (41.8%). The mean NIHSS score at hospital arrival was significantly higher in the lockdown period (9.4 ± 7.7 in 2020 vs. 8.4 ± 7.8 in 2019, *P* < 0.001). For the metrics measuring the quality of acute stroke care, the onset to door (OTD), onset to needle (ONT), and onset to recanalization (OTR) times didn't change significantly, while significant delays are shown for the door to CT scan (DTC, 1 min delay), door to needle (DTN, 4 min delays), and door to puncture (DTP, 29 min delays) times, which mainly happened in COVID-19 undesignated hospitals.

**Conclusions:** Profound reductions in stroke hospital admissions and significant delays in emergency care for acute ischemic stroke occurred during the pandemic of COVID-19. Engagement and effective communication with all stakeholders including patients, health care providers, governmental policymakers, and other implementation partners are required for future success in similar crises.

## Introduction

The coronavirus disease in 2019 (COVID-19) was declared a pandemic by the World Health Organization on 11th March 2020 (1). More than 200 countries have reported over 5 million cases and the disease progression is still ongoing. Because of the rapid spreading and clustering onset of this disease (2–4), it causes a major crisis to the whole healthcare system. Despite the stay-at-home orders in many countries, the incidence of other conditions is not diminishing, and rapid and effective medical care for serious and life-threatening conditions, such as stroke, is still paramount to achieve maximal functional recovery. To deliver timely and effective care with a balance to the risk of COVID-19 infectious exposure, the American Heart Association and American Stroke Association have issued series guides for stroke hyperacute management (5, 6). Similar academic societies in various regions and neurological physicians from different hospitals continue to make recommendations/guidelines and share treatment experiences (1, 7–10). Despite these efforts, acute stroke care is still negatively impacted (11–15). Reductions of 10–70% in stroke hospital admission are reported in several countries (13, 16–19), though COVID-19 may probably increase the risk of ischemic stroke as a result of endothelial injury and hypercoagulable status and other impacts on the central nervous system (20–22).

The adverse effect of the pandemic on stroke care is not only in quantity but also in quality. How much the emergency care for stroke has been affected during the prehospital and in-hospital stages in the lockdown period was still uncertain. In this study, we aimed to investigate the impact of the COVID-19 outbreak on stroke emergency care in Beijing (capital of China) during the pandemic. The results may provide valuable information to reorganize or optimize the current system of emergency care and to better prepare for a future pandemic.

## Methods

### Study Design

To contain the spread of COVID-19, the Chinese government launched the first-level response to major public health in 30 provinces from January to April. In Beijing, the lockdown period lasted for 97 days, with the same policies across all the administrative districts, from 01/24/2020 to 04/29/2020. We compare the stroke acute care using major stroke metrics in these 97 days with the same period in 2019.

### Data Collection

Since January 2018, an integrated database (the Beijing Emergency Care Database) connecting the emergency medical services (EMS) system and all the stroke centers was constructed in the city of Beijing by using a smartphone application (named “Green”). All emergency admissions for stroke were recorded in the database with a group of key metrics measuring the quality of acute stroke care, including time records for last known well, hospital arrival, images, intravenous thrombolysis (IVT), endovascular treatment (EVT), revascularization, and so on. In this study, the number of hospital admissions, as well as several quality measures for acute care of acute ischemic stroke (AIS), such as onset to door (OTD) time, door to CT scan (DTC) time, door-to-needle (DTN) time, door to puncture (DTP) time, onset to needle (OTN) time, onset to recanalization (OTR) time, were obtained from the database. The eligibility for IVT and EVT was in line with the recent guidelines (23, 24). During the lockdown period and the comparison period in 2019, there were 1,455 and 2,925 admissions recorded in the database, respectively. After deleting 174 and 571 patients in each period with diagnosis as other diseases or unknown, the remaining 1,281 and 2,354 stroke emergency admissions were analyzed in the study. To explore the correlation between stroke emergency hospital admissions and the number of newly diagnosed COVID-19 cases, the daily number of COVID-19 cases in Beijing was obtained from Beijing Municipal Health Commission (http://wjw.beijing.gov.cn/wjwh/ztzl/xxgzbd/gzbdyqtb/index_5.html).

### Statistical Analysis

The stroke hospital admissions in the lockdown period were compared with the numbers in the same period in 2019 and the percentage reduction was calculated. Descriptive statistics were used to compare the characteristics of stroke patients and the quality of care between two periods. Spearman correlation test was used to check the correlation between the number of weekly new diagnosed COVID-19 cases and the weekly stroke hospital admissions. The data on hospital admissions for stroke and COVID-19 cases were analyzed on a weekly basis. Quantitative variables were reported as mean (standard deviation, sd) or median (interquartile range, IQR). Categorical variables were represented as numbers and percentages. The differences between groups were tested using the Student *t*-test, Wilcox test, or Chi-square test. During the pandemic, 15 of 77 hospitals providing acute stroke care in Beijing were designated to receive patients sick with COVID-19. All the COVID-19 cases were sent to the 15 designated hospitals and received treatment there, while the remaining 62 undesignated hospitals did not treat any COVID-19 case. Therefore, all stroke admissions were divided into two groups according to whether the hospital was a designated COVID-19 hospital or not. Subgroup analyses were carried out by different types of hospitals (designated and undesignated). All analyses were conducted in R (V.3.6.3) (25).

## Results

### Number of Stroke Hospital Admissions

**Figure 1:**
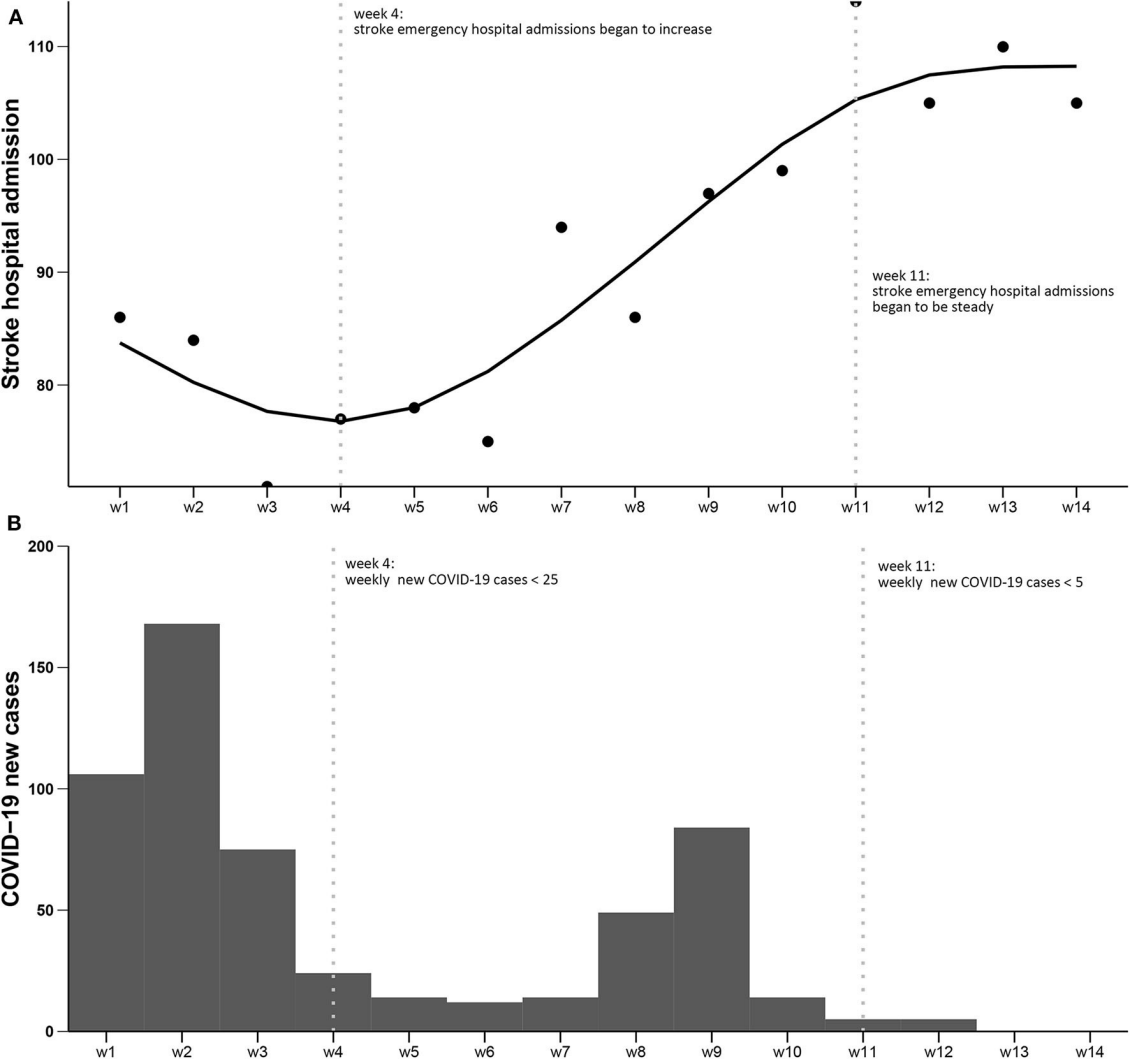
Numbers of stroke hospital admissions and numbers of COVID-19 cases from January 24th to April 29th, 2020 in Beijing. **(A)** Numbers of stroke hospital admissions; **(B)** Numbers of newly diagnosed COVID-19 cases. The two dotted vertical lines: (left) week 4, from February 14th, there were <25 newly diagnosed COVID-19 cases per week in the following month, and the stroke emergency hospital admissions began to increase; (right) week 11, from April 3rd, there were <5 newly diagnosed COVID-19 cases per week in the following month, and the stroke emergency hospital admissions reached a plateau.

From 24th January to 29th April 2020, there were 1,281 stroke hospital admissions in Beijing, this reflects a reduction of 45.6% compared with the same period in 2019 (2,354 admissions, Table 1). Compared with ischemic stroke (42.9%), there were more reductions for hemorrhagic stroke (69.0%). The reduction in emergency hospital admissions transferred by private car (48.1%) was slightly higher than transferred by ambulance (43.4%). More reductions happened in COVID-19 designated hospitals (52.6%) compared with undesignated hospitals (41.8%). The reduction in hospitals located in districts with fewer than 20 COVID-19 cases was similar to that in hospitals located in districts with 20 or more COVID-19 cases (Table 1). A negative correlation was shown between newly diagnosed COVID-19 cases and stroke emergency hospital admissions with marginal statistical significance (*r* = −0.176, *P* = 0.084). During the lockdown period, the number of stroke hospital admissions increased significantly from the 4th week and became steady from the 11th week (Figure 1A), which were the two points that newly diagnosed COVID-19 cases sharply dropped (Figure 1B).

**Table 1:** Effects of the pandemic on the Number of stroke admissions in Beijing.

	**Number of stroke admissions**		**Reductions (%)**
	**Comparison**	**Lockdown period**	
	**period in 2019**	**in 2020**	
All stroke admissions	2,354	1,281	45.6
Stroke types^*^			
Ischemic stroke	1,984	1,132	42.9
Hemorrhagic stroke	290	90	69.0
Other	80	59	26.3
Methods reaching to hospitals			
Ambulance	1,226	694	43.4
Private car	1,053	547	48.1
In-hospital stroke	75	40	46.7
COVID-19 designated hospitals^*^			
Yes	829	393	52.6
No	1,525	888	41.8
COVID-19 cases in the local district			
<20	771	417	45.9
≥20	1,583	864	45.4

* *The difference in distributions between two periods was statistically significant (P < 0.05)*.

### Characteristics of Stroke Patients

There were no significant differences in the mean age and sex distribution for stroke patients between two observational periods (Table 2). The NIHSS score of stroke patients at hospital arrival was significantly higher in the lockdown period (9.4 ± 7.7 in 2020 vs. 8.4 ± 7.8 in 2019, *P* < 0.001). The proportion of moderate stroke (NIHSS <6) was much lower, and the proportion of severe stroke (NIHSS >16) was much higher in the pandemic, especially in hospitals undesignated for COVID-19 (Table 2). For AIS, the proportions of patients receiving IVT or EVT therapy were significantly higher in the lockdown period when compared with the equivalent period in 2019 (Table 2).

**Table 2:** Main characteristics of stroke patients^*^.

	**All hospitals**			**Designated COVID-19 hospitals**			**Undesignated hospitals**		
	**Comparison period in 2019**	**Lockdown period in 2020**	***P*-value**	**Comparison period in 2019**	**Lockdown period in 2020**	***P*-value**	**Comparison period in 2019**	**Lockdown period in 2020**	***P*-value**
Age, y, mean ± sd	66.0 ± 13.3	65.7 ± 13.1	0.603	65.2 ± 12.6	64.2 ± 11.8	0.202	66.4 ± 13.6	66.4 ± 13.6	0.999
Sex, males, *N* (%)	1,583 (67.2)	854 (66.7)	0.750	546 (65.9)	270 (68.7)	0.358	1,037 (68.0)	584 (65.8)	0.279
Baseline NIHSS, mean ± sd	8.4 ± 7.8	9.4 ± 7.7	<0.001	7.5 ± 7.2	8.0 ± 6.5	0.225	8.9 ± 8.0	10.1 ± 8.2	0.002
Baseline NIHSS subgroups, *N* (%)^†^									
0–5	1,075 (47.0)	468 (40.3)	<0.001	441 (53.6)	175 (46.9)	0.066	634 (43.3)	293 (37.2)	0.001
6–16	886 (38.8)	501 (43.2)		286 (34.8)	155 (41.6)		600 (41.0)	346 (43.9)	
17–42	324 (14.2)	192 (16.5)		95 (11.6)	43 (11.5)		229 (15.7)	149 (18.9)	
Type of reperfusion therapy for AIS, *N* (%)			<0.001			<0.001			<0.001
IVT only	1,199 (60.4)	791 (69.9)		494 (66.5)	312 (80.6)		705 (56.8)	479 (64.3)	
EVT (with or without IVT)	250 (12.6)	185 (16.3)		80 (10.8)	55 (14.2)		170 (13.7)	130 (17.4)	
Not eligible for IVT or EVT	535 (27.0)	156 (13.8)		169 (22.7)	20 (5.2)		366 (29.5)	136 (18.3)	

* *AIS, acute ischemic stroke; IVT, intravenous treatment; EVT, endovascular treatment; NIHSS, NIH Stroke Scale/Score*.

† *With missing values*.

### Quality Metrics for AIS

**Figure 2:**
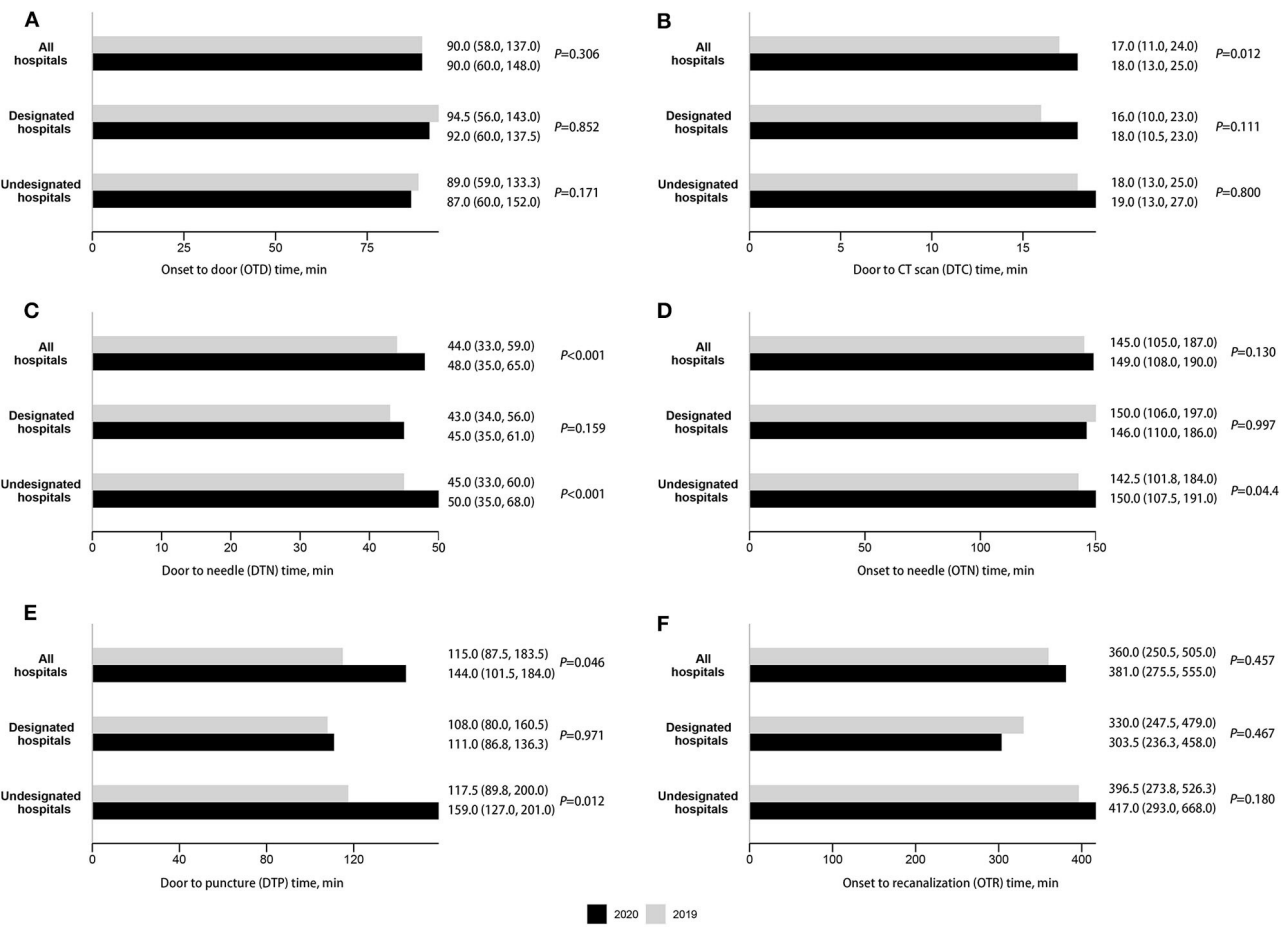
Time in workflows for the acute care provided to acute ischemic stroke (AIS), median (IQR). **(A)** Onset to door (OTD) time. **(B)** Door to CT scan (DTC) time. **(C)** Door to needle (DTN) time. **(D)** Onset to needle (OTN) time. **(E)** Door to puncture (DTP) time. **(F)** Onset to recanalization (OTR) time.

The time in workflow for AIS was shown in Figure 2 and Table 3. For the prehospital stage, there was no significant difference between the OTD times in two periods (Figure 2A). After hospital arrival, the DTC, DTN, and DTP times in the lockdown period were significantly longer than those in the equivalent period of 2019 (Figures 2B,C,E). The longer DTC, DTN, and DTP times in the lockdown period were shown in both COVID-19 designated and undesignated hospitals, but only the differences for DTN and DTP between two periods in undesignated hospitals were statistically significant (Figures 2B,C,E). During the whole emergency care workflow, there was no significant difference in the ONT and ORT times between two periods, except for the longer OTN time in undesignated hospitals during the pandemic (Figures 2D,F).

**Table 3:** Quality measures of the acute care provided for AIS^*^.

	**All hospitals**			**Designated COVID-19 hospitals**			**Undesignated hospitals**		
	**Comparison period in 2019**	**Lockdown period in 2020**	***P*-value**	**Comparison period in 2019**	**Lockdown period in 2020**	***P*-value**	**Comparison period in 2019**	**Lockdown period in 2020**	***P*-value**
TIME IN THE WORKFLOW, *N* (%)									
DTC time									
DTC ≤25 min	923 (78.8)	596 (75.9)	0.150	389 (81.0)	241 (84.0)	0.331	534 (77.2)	355 (71.3)	0.022
IVT only									
DTN time									
DTN ≤45 min	552 (53.4)	307 (45.8)	<0.001	239 (55.7)	129 (52.2)	0.423	313 (51.8)	178 (42.1)	0.002
DTN ≤60 min	814 (78.8)	473 (70.6)	<0.001	345 (80.4)	180 (72.9)	0.027	469 (77.6)	293 (69.3)	0.003
OTN time									
OTN ≤3 h	751 (72.7)	482 (71.9)	0.739	303 (70.6)	181 (73.3)	0.480	448 (74.2)	301 (71.2)	0.286
OTN ≤3.5 h	854 (82.7)	544 (81.2)	0.438	340 (79.3)	200 (81.0)	0.620	514 (85.1)	344 (81.3)	0.124
OTN ≤4.5 h	986 (95.5)	640 (95.5)	1.000	407 (94.9)	232 (93.9)	0.602	579 (95.9)	408 (96.5)	0.744
EVT (with or without IVT)									
DTP time									
DTP ≤90 min	43 (30.9)	23 (20.0)	0.061	19 (37.3)	12 (30.0)	0.511	24 (27.3)	11 (14.7)	0.057
OTR time									
OTR ≤24 h	133 (95.7)	112 (97.4)	0.518	50 (98.0)	40 (100.0)	1.000	83 (94.3)	72 (96.0)	0.727
THERAPY OUTCOME									
IVT only									
NHISS after acute care, mean ± sd	5.2 ± 5.8	6.4 ± 6.5	<0.001	4.7 ± 5.6	6.1 ± 6.3	0.002	5.5 ± 6.0	6.5 ± 6.7	0.011
NHISS after acute care, ≤1, *N* (%)	149 (14.4)	75 (11.2)	0.057	77 (17.9)	31 (12.6)	0.081	72 (11.9)	44 (10.4)	0.484
EVT (with or without IVT)									
NHISS after acute care, mean ± sd	11.3 ± 8.9	11.9 ± 9.3	0.583	10.7 ± 7.1	11.5 ± 8.4	0.608	11.6 ± 9.8	12.1 ± 9.9	0.768
NHISS after acute care, ≤1, *N* (%)	20 (14.4)	14 (12.2)	0.712	4 (7.8)	6 (15.0)	0.325	16 (18.2)	8 (10.7)	0.192
Recanalization, *n* (%)	133 (95.7)	115 (100.0)	0.034	49 (96.1)	40 (100.0)	0.502	84 (95.5)	75 (100.0)	0.125

* *AIS, acute ischemic stroke; IVT, intravenous treatment; EVT, endovascular treatment; NIHSS, NIH Stroke Scale/Score; DTC, door to CT scan; DTN, door to needle; OTN, onset to needle; DTP, door to puncture; OTR, onset to recanalization*.

In line with the longer DTN time in the lockdown period, the proportions of DTN ≤45 min and DTN ≤60 min decreased significantly in both designated and undesignated hospitals, except for the proportion of DTN ≤45 min in designated hospitals (Table 3). It is noteworthy that despite the longer DTC and DTP time in the pandemic, the proportions of DTC ≤25 min and DTP ≤90 min didn't change significantly, except for the drop in the proportion of DTC ≤25 min in undesignated hospitals (Table 3). Besides, the proportions of OTN ≤3, 3.5, 4.5, and OTR ≤24 h didn't change significantly in both designated and undesignated hospitals (Table 3).

### Clinical Outcomes

For AIS patients who received IVT, although the mean NIHSS score after therapy was significantly higher in the lockdown period, the difference in the proportion of patients with NIHSS ≤1 between two periods was not statistically significant (Table 3). For AIS patients who received EVT, the differences in the mean NIHSS scores after therapy and the proportion of patients with NIHSS ≤1 between two periods were both not statistically significant (Table 3). The recanalization rate in the lockdown period was significantly higher than that in the comparison period of 2019 (Table 3).

## Discussion

Based on records from all stroke centers in Beijing, we found that during the 97 days of the lockdown period, the number of stroke emergency hospital admissions reduced significantly by almost 50% compared with the equivalent period in 2019. There were delays in DTC, DTN, and DTP times for AIS, which mostly occurred after stroke patients arrived in the emergency room in COVID-19 undesignated hospitals.

During the pandemic, one major negative impact on acute care for stroke was the decline in emergency hospital admissions, consistent with reports in different regions in China or other countries. The degree of reduction varied from 10 to 70% (11, 13, 13, 16–19, 26–31). One recent report in China also reported a 40% reduction in stroke hospital admissions (16), similar to our results (45.6%). Similar hospitalization reduction was also observed in patients with acute coronary syndrome (32). Several reasons may contribute to the declines, the most possible one may be that stroke patients and their families didn't come to hospitals because of the social distance requirement and the concerns for in-hospital cross-infection (16). Patients with mild stroke symptoms may choose not to come to the hospital. In our data, patients with NIHSS <6 dropped by nearly 7% (47.0 vs. 40.3%). According to our data, we can see a negative correlation between the newly diagnosed COVID-19 cases and stroke admissions in the lockdown period, though the correlation was not statistically significant. Besides, a concordance of the decrease in newly diagnosed COVID-19 cases and the increase in stroke admissions was shown in the 4th week during the lockdown period. This may indirectly reflect the severity of the pandemic related to stroke patients' behavior in seeking emergency care during this crisis. Thus, an early rapid response to COVID-19 and early control of disease could be critical to mitigating the negative impact on other diseases, such as stroke, requiring emergency care. On the other hand, during the pandemic, patients with life-threatening conditions should be encouraged to seek medical services and be assured that efforts were made by hospitals to prevent in-hospital cross-infection.

More declines were seen in hospitals designated for COVID-19 than in undesignated hospitals. Reasons from two aspects may contribute to this result. For reasons from patients and their families, they may reluctant to bother hospitals busy with taking care of COVID-19 cases and be fear of getting infected in designated hospitals (33). For reasons from the stroke emergency care system, more stroke patients who called the emergency centers may be recommended to undesignated hospitals purposely for appropriate allocation of health care resources. Our data showed that for stroke patients transferred by ambulance in the lockdown period, there were 22.3% (155/694) and 77.7% (539/694) of them sent to designated and undesignated hospitals, respectively, while the percentages were 41.9% (229/547) and 58.1% (318/547) for patients who reached hospitals by private cars. A recent survey in China also reported the capacity for stroke care reduced in most COVID-19 designated hospitals (16).

Our results showed more reductions for hemorrhagic stroke (69.0%) compared with ischemic stroke (42.9%). While we do not have a clear answer to explain the discrepancy, we speculate that staying-at-home makes patients more likely to take medication which subsequently may better control the blood pressure and reduced the incidence of hemorrhagic stroke.

We didn't observe a constant rate of reduction in admission with moderate and severe strokes. According to the results, the baseline NIHSS score for patients in the lockdown period was significantly higher than the comparable period. This was similar to previous report (29). Patients with mild symptoms may reluctant to seek medical care with concerns of COVID-19. Patients were advised to cancel or postpone some non-essential medical procedures in the lockdown period. However, for patients with stroke, whether they need an emergency intervention or not requires the assessment by professional neurologists but not by themselves. Therefore, in this situation, improving the awareness of the importance of stroke emergency care in general, especially in the high-risk population was paramount.

Our study also examined the question whether there was a delay in emergency care for AIS. In the previous reports, significant delays happened in prehospital stage (14, 34), but whether there were delays in workflow metrics during the acute care was conflict (13, 14, 26, 28). According to our results, in the prehospital stage, the OTD time became shorter in the lockdown period, which may benefit from the better traffic situation. The delay happened after hospital arrival, that the DTC, DTN, and DTP time became longer in the lockdown period. This may because of the protected workflow in a pandemic crisis, including an additional screen for infection and the use of personal protection equipment (8). Despite the longer DTC and DTP time, the percentages of patients with DTC ≤25 min and with DTP ≤90 min didn't drop significantly. Besides, the OTN and OTR time, which reflect the whole emergency care workflow, were not increased significantly during the lockdown period. These results reflected a relatively efficacious stroke emergency care system in Beijing. Even though, the percentage of patients with DTN ≤45 min and DTN ≤60 min were dropped in the pandemic. Further optimization in the stroke emergency care system is needed.

A discrepancy in two types of hospitals was shown that the delays mostly happened in undesignated hospitals. We think the possible reason is that in COVID-19 designated hospitals, the more reductions for all kinds of patients may reduce the transfer and intersect time between departments. Moreover, the health providers in these hospitals were well-trained to avoid in-hospital infections because of COVID-19 cases in their hospitals. They may be more familiar with the protected stroke care workflow than providers in undesignated hospitals. If this is true, strengthed training and practice programs for protected workflows in health providers in undesigned hospitals may reduce the delays in stroke emergency care or even other medical procedures. To find the reason clearly, further investigations are needed to compare the relevant details in two kinds of hospitals. Such researches are valuable to improve the whole stroke emergency care system in a region and make it less vulnerable to a similar crisis in the future.

There were several limitations in this study. Firstly, during the lockdown period, most of AIS patients had severe symptoms, and we may expect worse prognosis outcomes. Because of the limited variables we got, the outcomes for AIS emergency care should be evaluated in more detail by further studies. Detail information for etiology types and vessel occlusion sites were also not recorded, we cannot estimate the impact on acute care of stroke with different aetiological types or with different vessel occlusion sites. Secondly, our database did not record the status of COVID-19 and is unable to examine the potential association of COVID-19 and stroke. However, there were only about 600 reported COVID-19 cases in Beijing during the lockdown period, and the number is relatively small. Thirdly, as there was a suspicion on the association between COVID-19 infection and the risk of stroke, as well as the medical resource encroachment from COVID-19 cases, the influence on stroke emergency care in regions with higher infection rates may differ. Fourth, we chose the same period in 2019 as the comparison group mainly because of the seasonal fluctuation in stroke hospital admissions reported previously (35). However, there was also an increasing secular trend in stroke hospital admissions and a consistent improvement in the acute care process which result in decreases in therapy time. We could not exclude the possibility that the estimation of the impact was biased downward. Thus, the interpretation of these results should be careful. Even though, we can still see a significant negative effect on stroke emergency care in a region with a low infection rate.

In conclusion, our study demonstrated a major reduction of stroke hospitalization admissions in the lockdown period in Beijing. There were delays in acute care for AIS, and there are differences between COVID-19 designated hospitals and undesignated hospitals. These results have important implications to better prepare acute stroke care in future pandemics like this. Engagement and effective communication with all stakeholders including patients, health care providers, governmental policymakers, and other implementation partners are required for future success.

## Data Availability Statement

Summarized data can be shared by request from any qualified investigator to the correspondence author.

## Ethics Statement

The study was reviewed and approved by Ethics Committee of Xuan Wu Hospital, Capital Medical University. Written informed consent for participation was not required for this study in accordance with the national legislation and the institutional requirements.

## Author Contributions

HS, YWu, YWa, and YL contributed to the study concept and had full access to all the data in the study. YWu and ZW responsibility for the integrity of the data and interpreted the findings. YWu and FC contributed to data analysis and drafted the article. WF and YWa interpreted the data. All authors contributed to the critical revision of the article for important intellectual content and agreed to the final version of the manuscript. YWa and HS attests that all listed authors meet authorship criteria and that no others meeting the criteria have been omitted.

## Conflict of Interest

The authors declare that the research was conducted in the absence of any commercial or financial relationships that could be construed as a potential conflict of interest.
